# Development of a novel moisture-suppression bag for the preservation of hygroscopic medications

**DOI:** 10.1186/s40780-022-00273-8

**Published:** 2023-02-15

**Authors:** Taisuke Matsuo, Takayuki Yoshida, Kiyotaka Ushijima, Takashi Doi, Yasuyuki Sadzuka, Takashi Tomita

**Affiliations:** 1 Division of Advanced Pharmaceutics, Department of Clinical Pharmaceutical Sciences, School of Pharmacy, Iwate Medical University, 1-1-1 Idaidori, Yahaba-cho, Shiwa-gun, Iwate, 028-3694 Japan; 2 Division of Pharmacy Function assessment, Faculty of Pharmaceutical Sciences, Teikyo Heisei University, 4-21-2, Nakano, Nakano-ku, Tokyo, 164-8530 Japan; 3 Development Department, Maruto Sangyo Co., Ltd., 892-1, Hikata, Ogori, Fukuoka, 838-0112 Japan; 4 Department of Pharmaceutical Sciences, Faculty of Pharmaceutical Sciences, Teikyo Heisei University, 4-21-2, Nakano, Nakano-ku, Tokyo, 164-8530 Japan

**Keywords:** One-dose package, Hygroscopic medication, Moisture absorption, Moisture-suppression bag, Desiccating agent

## Abstract

**Background:**

One-dose packaging is frequently used in Japan for elderly patients who are prescribed several medications. It is useful for easy administration and the prevention of misuse or missed medications. Hygroscopic medications are not suitable for one-dose packaging because moisture absorption may alter their properties. Plastic bags with desiccating agents are sometimes used to store hygroscopic medicines in one-dose packaging. However, the relationship between the quantity of desiccating agents and their safety in the storage of hygroscopic medications is poorly understood. Furthermore, older adults might accidentally consume desiccating agents used in food preservation. In this study, we developed a bag that suppresses the moisture absorption of hygroscopic medications without the use of desiccating agents.

**Methods:**

The bag was manufactured using polyethylene terephthalate, polyethylene, and aluminum film on the outside, and unified with a desiccating film on the inside.

**Results:**

The relative humidity (RH) in the bag was maintained at approximately 30–40% when the bag was stored at 75% RH and 35 °C. The manufactured bag’s moisture suppressing effect was better than that of plastic bags with desiccating agents when the hygroscopic medications, potassium aspartate and sodium valproate tablets, were stored at 75% RH and 35 °C for 4 weeks.

**Conclusions:**

The moisture-suppression bag effectively stored and preserved hygroscopic medications and was more effective in inhibiting moisture absorption than plastic bags with desiccating agents under high temperature and humidity conditions. The moisture-suppression bags are expected to be useful for elderly patients who are prescribed several medications in one-dose packaging.

## Background

One-dose packaging of medications is an important aspect of pharmaceutical care in Japan. It is usually employed for elderly patients to allow for easy administration of medication and prevent misuse and missed medications [1, 2]. Cellophane polyethylene laminating paper and glassine paper are currently used as components for one-dose packaging. However, these materials have higher water permeability than press-through packaging and strip packaging. Therefore, the storage of hygroscopic medications in one-dose packaging is not appropriate because moisture absorption leads to a decrease in their therapeutic potential and a deterioration of their physical properties, such as changes in their external appearance and a reduction in hardness.

When hygroscopic medications are dispensed as one-dose packs, the storage of these medications with desiccating agents is important to retain their stability and efficacy [3, 4]. However, the relationship between the quantity of desiccating agents and their safety when used in medication storage is poorly understood. In addition, it has been reported that elderly persons eat desiccating agents included in food products by mistake [5, 6]. One-dose packaging is frequently used for medications dispensed to elderly patients, especially when numerous medications are prescribed; therefore, storage methods without the use of desiccating agents are desirable from a safety standpoint. In this study, a moisture-suppression bag without desiccating agents was developed, and its effectiveness in storing hygroscopic medications was compared with that of a plastic bag containing desiccating agents.

## Methods

### Moisture-suppression bag

**Fig. 1:**
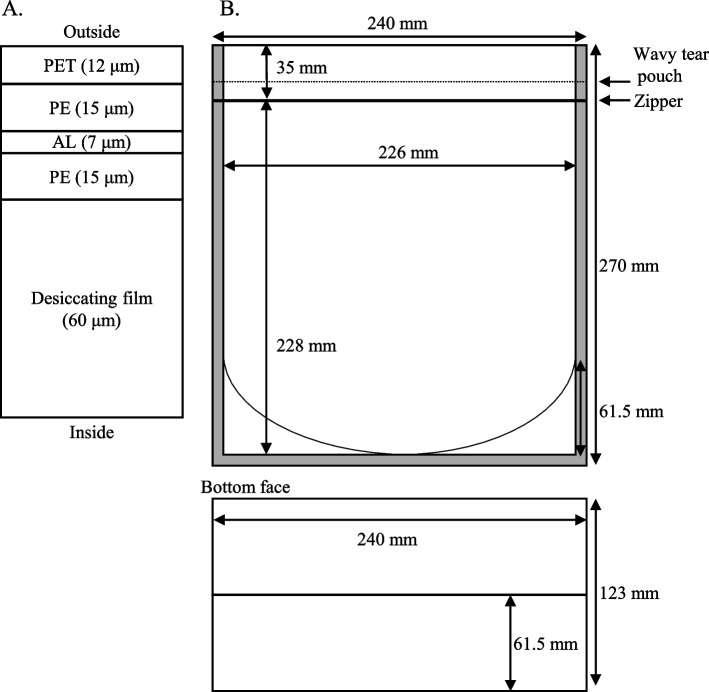
Blueprint of moisture-suppression bag. **A** Unified polyethylene terephthalate (PET), polyethylene (PE), and aluminum (AL) film on the outside, and a desiccating film on the inside. **B** Design of the moisture-suppression bag

**Fig. 2:**
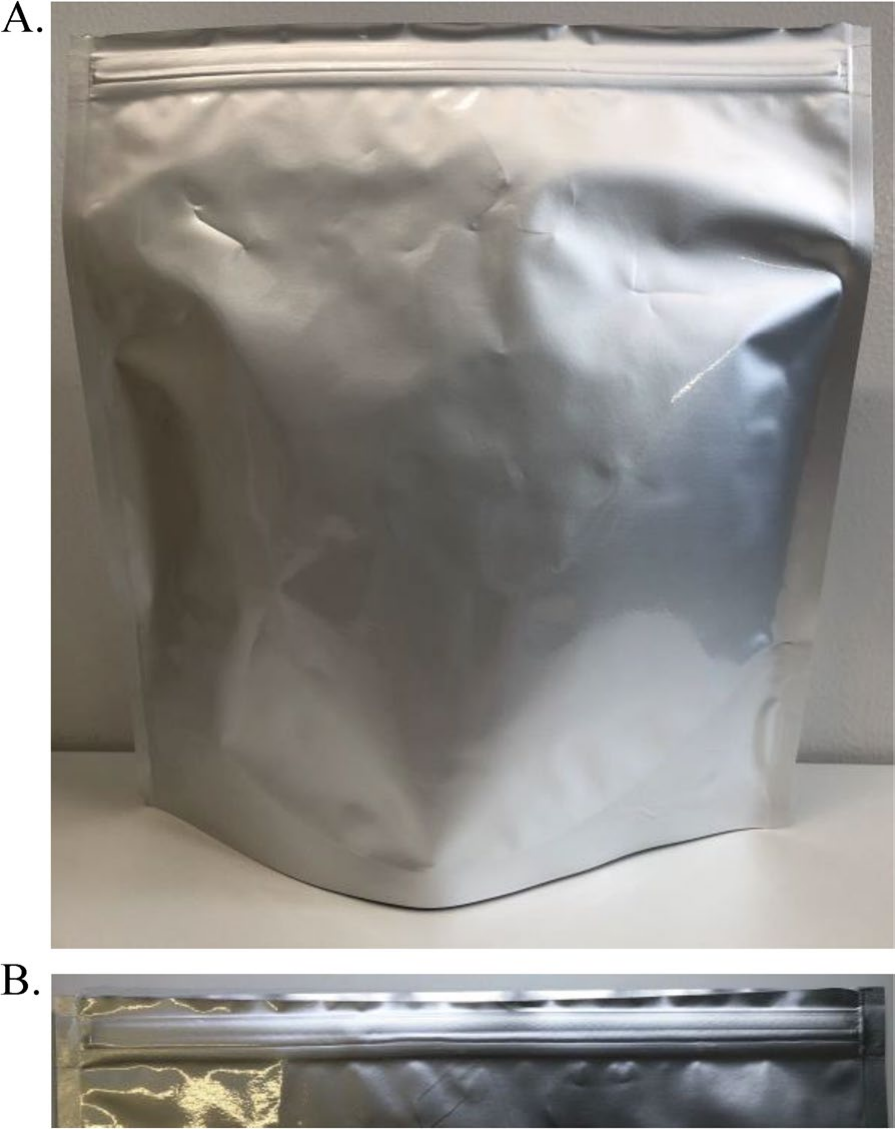
Appearance of the moisture-suppression bag. **A** The appearance of the moisture-suppression bag and **B** the wavy tear pouch after opening are shown

We developed a moisture-suppression bag using polyethylene terephthalate, polyethylene, and aluminum films on the outside and unified with a desiccating film on the inside (Kyushitsu-kun, Maruto Sangyo Co., Ltd., Fukuoka, Japan) (Fig. 1A). The bag has the following characteristics: (i) A standing zip-type bag with dimensions of 240 mm (width) × 270 mm (height) × 61.5 mm (thickness) (Fig. 1B, 2A) and adequate internal storage dimensions of 226 mm (width) × 228 mm (height) × 61.5 mm (thickness) to store frequently used medicines in Japan. (ii) An aluminum film layer to suppress water vapor permeability and the penetration of light. (iii) A wavy tear pouch (Tsukameru-kun in Japanese, patent number in Japan: 6966621) (Fig. 2B) to allow for easy opening, particularly convenient for the elderly. (iv) The outside of the bag can be labeled according to the individual requirements of facilities, such as hospitals and pharmacies.

### Selection of hygroscopic medication

Hygroscopic medications were selected from the “Project to Collect and Analyze Pharmaceutical Near-Miss Event Information” database of the Japan Council for Quality Health Care. This database collects and analyzes near-miss events reported by pharmacies. Hygroscopic medications were selected according to the following criteria and outcomes: (i) The data collection period was from April 2009 to May 2020 (the total number of cases was 326,111); (ii) The keywords one-dose packaging and/or moisture absorption were applied, and 116 search results were obtained with 102 cases of reported medications that had problems with one-dose packaging. As one case reported two medications, the total number of reported medications was 103. Problems with potassium aspartate tablets (16 cases) and sodium valproate tablets (15 cases) were the most frequently reported. Consequently, these medications were selected for this study.

### Measurement of relative humidity (RH) in moisture-suppression bags and plastic bags with desiccating agents

RH was measured using a button-type temperature and humidity data logger, “Hygrochron” (KN Laboratories, Inc., Osaka, Japan), as previously described [7]. The logger was packaged in cellophane polyethylene laminating paper 20 μm thick (Yuyama Co., Ltd., Osaka, Japan) and stored in a moisture-suppression bag or plastic bag with desiccating agents (15 or 30 g). Silica gel desiccant (7.5 g/pack, Daiso Industries Co., Ltd., Hiroshima, Japan) was used as the desiccating agent because calcium oxide, which is also a desiccating agent, causes chemical burns when mistakenly consumed [5, 6, 8, 9]. These bags were stored at 75% RH and 25 °C or 35 °C (Chamber, Constant Temperature and Humidity IG421, Yamato Scientific Co., Ltd., Tokyo, Japan) for 28 days. The RH in each package was measured every 3 h (*n* = 3). The RH in the external environment was measured in the absence of bags (*n* = 2). The results of this experiment were shown from 3 to 168 h.

### Evaluation of the quality of hygroscopic medications under storage

ASPARA Potassium Tablets 300 mg (lot: E381A, Nipro ES Pharma Co., Ltd., Osaka, Japan) and Depakene Tablets 200 mg (lot: 907AJE, Kyowa Kirin Co., Ltd., Tokyo, Japan) were used as potassium aspartate and sodium valproate tablets, respectively. The medications were packaged in cellophane polyethylene laminating paper (1 tablet/package). The samples were stored in a moisture-suppression bag or plastic bag with a desiccating agent (15 or 30 g). The storage conditions were 75% RH and 25 °C or 35 °C for 28 days (*n* = 5). The bags were opened when performing the analyses. As the control, the storage of medications packaged in cellophane polyethylene laminating paper without the bags was performed under the same conditions. The analyses were performed for 7 days.

### Statistical analysis

The results are shown as mean ± standard deviation. Statistical analyses were performed using the student’s *t*-test or one-way ANOVA with the Tukey–Kramer test. Statistical significance was set at *P* < 0.05.

## Results

### RH conditions in the moisture-suppression bag and plastic bags with desiccating agents

**Fig. 3:**
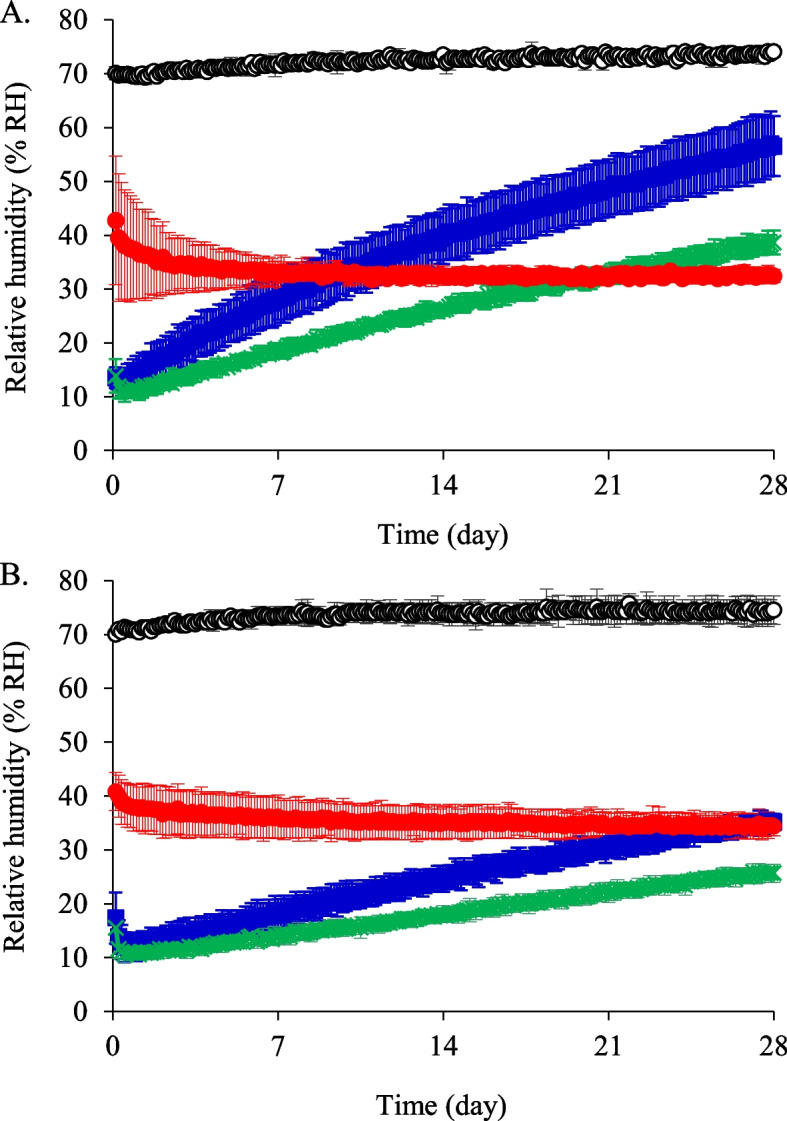
Relative humidity in the moisture-suppression bag and the plastic bags with desiccating agents (15 or 30 g). The bags were stored at 75% RH and 35 °C (**A**) or 25 °C (**B**).  : external environment (*n* = 2),  : moisture-suppression bag (*n* = 3),  : plastic bag with 15 g desiccating agent (*n* = 3),  : plastic bag with 30 g desiccating agent (*n* = 3)

The moisture-suppression bag and plastic bags with desiccating agents were stored at 75% RH and 35 °C or 25 °C for 28 days. When the moisture-suppression bag was stored at 75% RH and 35 °C, the RH at 3 h was 43%, which decreased and was maintained at approximately 32–35% for 28 days (Fig. 3A). However, the minimum values of RH in the plastic bags with desiccating agents (15 or 30 g) were 13% at 6 h and 11% at 9 h. These values increased in a time-dependent manner, and the values at 28 days were 39 and 33% for the 15 g and 30 g desiccating agents, respectively (Fig. 3A). These values were higher than those observed for the moisture-suppression bag.

When the bags were stored at 25 °C and 75% RH, the RH in the moisture-suppression bag was similar to that observed when stored at 35 °C and 75% RH (Fig. 3B). However, the RH values in the plastic bags with desiccating agents at 25 °C and 75% RH were lower than those of the same bags when stored at 35 °C and 75% RH. On day 28, the RH in the external environment was 75%, and that in the moisture-suppression bag and the plastic bags with 15 g and 30 g desiccating agents was 34, 35, and 26%, respectively (Fig. 3B).

### Quality of potassium aspartate tablets stored in moisture-suppression bags at 35 °C or 25 °C and 75% RH

**Fig. 4:**
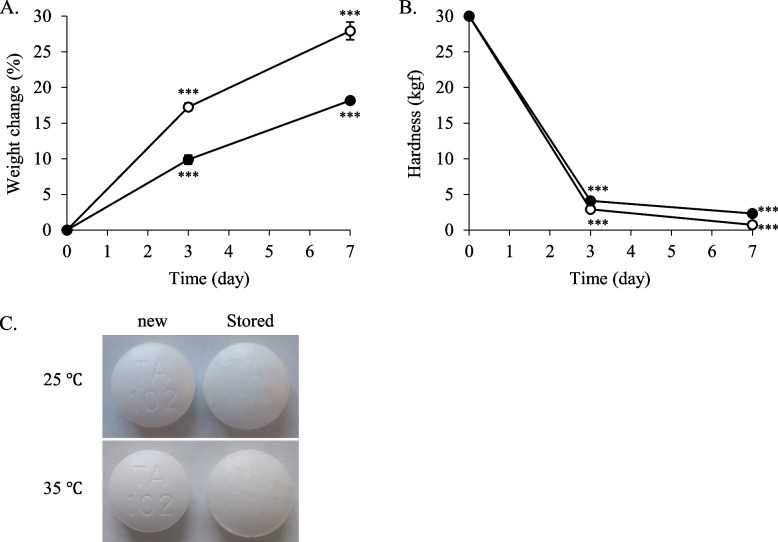
Quality changes of potassium aspartate tablets packaged in cellophane polyethylene laminating paper. The tablets were stored at 75% RH and 25 °C or 35 °C. Weight change (*n* = 5) (**A**), hardness (*n* = 5) (**B**), and appearance (**C**) were examined. **A**, **B**  : stored at 35 °C;  : stored at 25 °C. ^***^P < 0.001 (vs. day 0, *t*-test). **C** The appearance at day 7 is shown

**Fig. 5:**
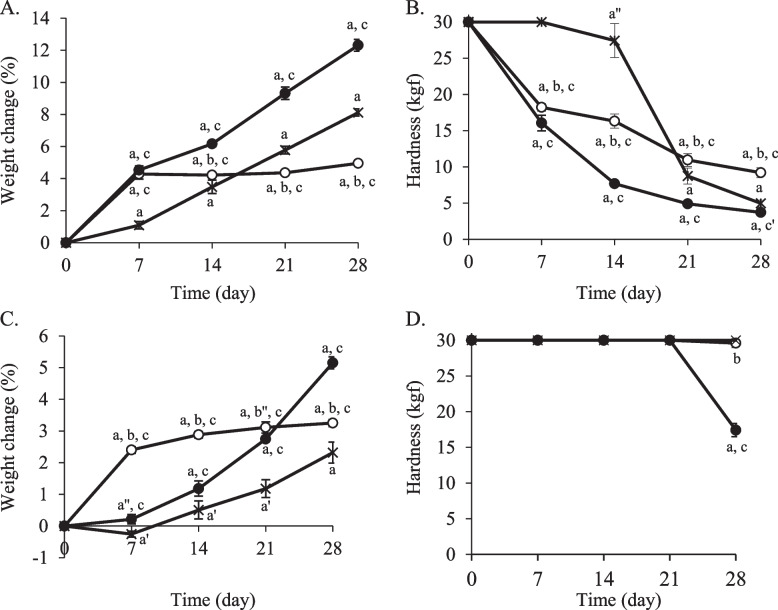
Quality changes of potassium aspartate tablets stored in the moisture-suppression bag. The tablets packed in cellophane polyethylene laminating paper were stored in the moisture-suppression bag and plastic bags with 15 or 30 g desiccating agents. The samples were stored at 75% RH and 35 °C (**A**, **B**) or 25 °C (**C**, **D**). Weight change (*n* = 5) (**A**, **C**) and hardness (*n* = 5) (**B**, **D**) are shown.  : moisture-suppression bag,  : plastic bags with 15 g desiccating agent, ×: plastic bags with 30 g desiccating agent. ^a^*P* < 0.001, ^a′^*P* < 0.01, ^a″^*P* < 0.05 (vs. day 0), ^b^*P* < 0.001, ^b″^*P* < 0.05 (vs. plastic bags with 15 g desiccating agent), ^c^*P* < 0.001, ^c′^*P* < 0.01 (vs. plastic bags with 30 g desiccating agent) (Tukey–Kramer test)

The hardness of the potassium aspartate tablets immediately after they were taken from the press-through packaging was over 30 kgf. The weight changes of the potassium aspartate tablets, packaged in cellophane polyethylene laminating paper and stored at 35 °C and 75% RH for 3 and 7 days, increased by 17 and 28%, respectively (Fig. 4A). The hardness of the tablets stored for 3 and 7 days decreased by approximately 3 kgf and 1 kgf, respectively (Fig. 4B). Furthermore, the coating film on the surface of the tablets stored for 7 days had peeled off (Fig. 4C). Under the same conditions, when the tablets were stored in the moisture-suppression bag, the weight increased by 4% in 7 days (Fig. 5A). The weights were almost maintained for 28 days, with a weight change of 5% observed at 28 days. When the tablets were stored in a plastic bag with a 15 g desiccating agent, the weight change at 7 days was 5%, which increased in a time-dependent manner, and the weight change at 28 days was 12% (Fig. 5A). In the case of storage with a 30 g desiccating agent, the weight changes for the first 14 days were lower than those observed following storage in the moisture-suppression bag; however, from 21 days, the observed weights were higher than those from the moisture-suppression bag. The observed weight change at 28 days was 8% (Fig. 5A). The hardness of the tablets stored in the moisture-suppression bags decreased in a time-dependent manner and was 9 kgf at 28 days (Fig. 5B). Following the use of plastic bags with a 15 g desiccating agent, the hardness was lower than that of the tablets stored in the moisture-suppression bags. In plastic bags with a 30 g desiccating agent, the hardness of the tablets was almost maintained for 14 days; however, it significantly decreased from 21 days, with a 5 kgf hardness at 28 days (Fig. 5B).

When the potassium aspartate tablets were packaged in cellophane polyethylene laminating paper and stored at 25 °C and 75% RH for 7 days, the weight and hardness changes at 3 or 7 days were a 10% or 18% increase and a 4 or 2 kgf decrease, respectively (Fig. 4A, B). After 7 days, the coated film of the tablets had peeled off (Fig. 4C). Under the same conditions, the weight changes of the potassium aspartate tablets stored for 28 days in the moisture-suppression bag and those stored with the 15 g and 30 g desiccating agents were 3, 5, and 2%, respectively (Fig. 5C). The hardness in all cases was maintained for 21 days (Fig. 5D). At 28 days, the hardness of the tablets stored with the 30 g desiccating agent was maintained. Contrastingly, the hardness of the tablets stored in the moisture-suppression bag and with the 15 g desiccating agent had decreased to 29.6 kgf and 17 kgf, respectively (Fig. 5D).

### Quality of sodium valproate tablets stored in moisture-suppression bags at 35 °C or 25 °C and 75% RH

**Fig. 6:**
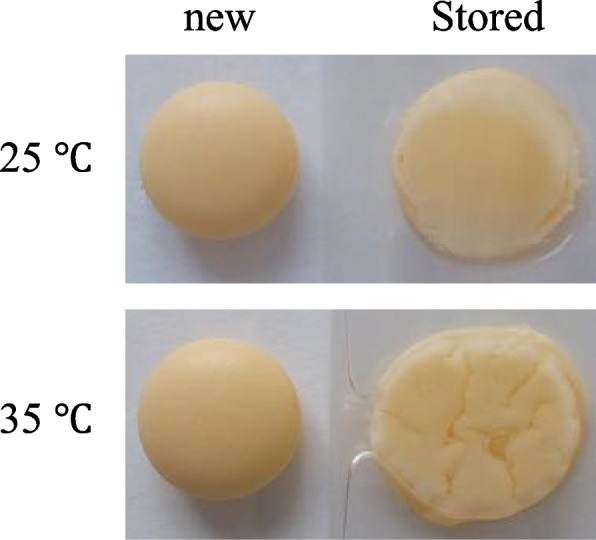
Appearance of sodium valproate tablets packaged in cellophane polyethylene laminating paper. The tablets were stored at 75% RH and 25 °C or 35 °C. The appearance at day 7 is shown

**Fig. 7:**
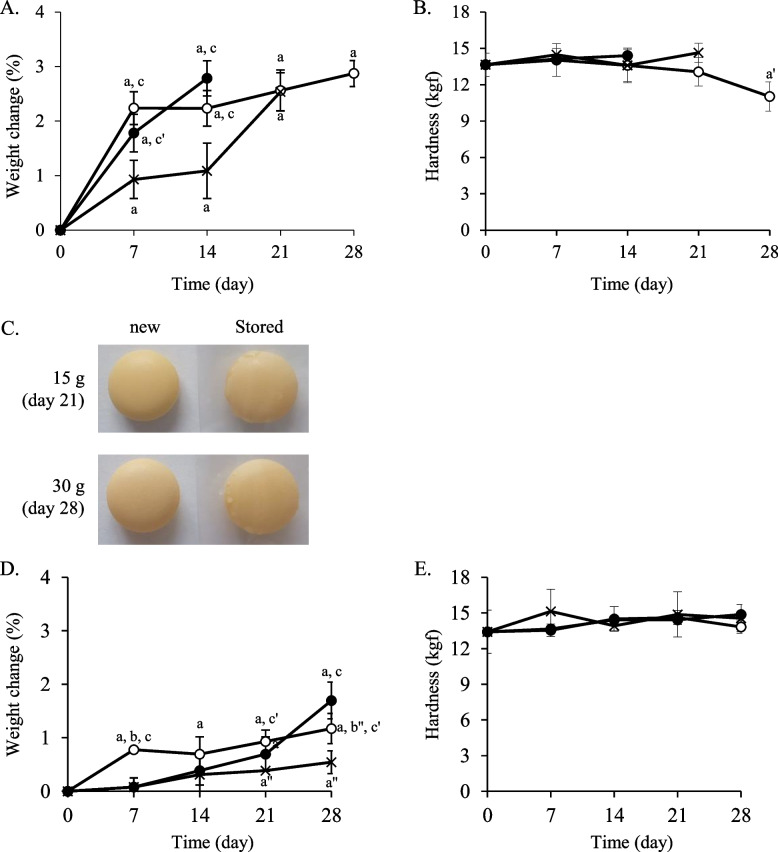
Quality changes of sodium valproate tablets stored in the moisture-suppression bag. The tablets packed in cellophane polyethylene laminating paper were stored in the moisture-suppression bag and plastic bags with 15 or 30 g desiccating agents. The samples were stored at 75% RH and 35 °C (**A**–**C**) or 25 °C (**D, E**). Weight change (*n* = 5) (**A**, **D**), hardness (*n* = 5) (**B**, **E**), and appearance (**C**) are shown.  : moisture-suppression bag,  : plastic bags with 15 g desiccating agent, ×: plastic bags with 30 g desiccating agent. ^a^*P* < 0.001, ^a′^*P* < 0.01, ^a″^*P* < 0.05 (vs. day 0), ^b^*P* < 0.001, ^b″^*P* < 0.05 (vs. plastic bags with 15 g desiccating agent), ^c^*P* < 0.001, ^c′^*P* < 0.01 (vs. plastic bags with 30 g desiccating agent) (Tukey–Kramer test)

Under 75% RH condition, the sodium valproate tablets stored at both 35 °C and 25 °C in the control group were observed to gradually deliquesce on day 1 (data not shown), and therefore, weight and hardness could not be measured. Further, they had completely lost their physical tablet-like appearance by day 7 (Fig. 6). At 35 °C, although the tablets stored in the moisture-suppression bags absorbed moisture from day 7 (Fig. 7A), the hardness was maintained for 21 days. The hardness decreased after 28 days, but the value was over 10 kgf (Fig. 7B). Furthermore, the tablets did not deliquesce until day 28. Conversely, the tablets stored in plastic bags with the 15 g and 30 g desiccating agents showed deliquescence at 21 and 28 days, respectively (Fig. 7C). Therefore, their weight and hardness were not measured. At 25 °C, the weight changes of the sodium valproate tablets stored in the moisture-suppression bag and those stored with the 15 g and 30 g desiccating agents at day 28 were 1.2, 1.7, and 0.5%, respectively (Fig. 7D). The hardness in all cases did not decrease during the 28-day observation period (Fig. 7E).

## Discussion

Appropriate storage of hygroscopic medications in one-dose packaging is crucial in clinical practice [3, 7, 10]. Under the same RH, when temperature is high, moisture absorption by hygroscopic medications increases according to a rise in humidity in the bags. Our moisture-suppression bags maintained 30–40% RH, adequately preserving hygroscopic medications when stored at 25 °C or 35 °C and 75% RH. Regarding desiccating agents, the dehumidification ability of the 30 g agent was stronger than that of the 15 g agent. It is thus desirable to use more desiccating agents under high temperature and high humidity conditions. The RH in the plastic bags with the 30 g desiccating agent was lower than that observed in the moisture-suppression bag for a while. However, the RH increased in a time-dependent manner and was higher than that in the moisture-suppression bag when the bags were stored at 35 °C and 75% RH for 4 weeks. Therefore, the qualities of the medications in the moisture-suppression bag were maintained compared to those of medications stored in the bag with desiccating agents under high temperature and high humidity conditions. However, in this study, the bags were opened every week or after 4 weeks for analyses; therefore, the observed values were lower than what would be observed in real-life clinical settings. In cases of therapeutic regimens, such as those that require dosage form administration three times/day, moisture absorption would be further increased compared with our experimental findings.

In clinical practice, plastic bags with desiccating agents are sometimes used for one-dose packaging. Desiccating agents of silica gel (size: 1.0–2.0 g) are typically included in the aluminum packing of new hygroscopic medications. Such desiccating agents are not considered adequate to safely store hygroscopic medications in one-dose packaging. In addition, desiccating agents are dangerous for the elderly and patients with schizophrenia or dementia, who may eat them by mistake [5, 6, 8, 9]. Therefore, the moisture-suppression bags would be useful for these patients. In clinical practice, it is necessary for healthcare workers, such as pharmacists, to decide when to use the moisture-suppression bags or bags with desiccating agents according to the conditions and preferences of patients.

In Japan, containers and packaging are manufactured according to the “Specifications and Standards for Food, Food Additives, etc. Ministry of Health and Welfare Notification No. 370, 1959”. Our moisture-suppression bag was also manufactured according to these specifications and standards, and therefore, the bag is considered safe. However, sensitivity to the bag’s smell differs among people, making it necessary for patients to assess these bags for smell, storage capability, usability, and effective preservation of each hygroscopic medication in their individual real-world settings. In this study, two hygroscopic medications were examined to clarify the efficiency of the developed moisture-suppression bag. The type of hygroscopic medication and its preservation time are very crucial factors to consider for storing one-dose packaged medications in moisture-suppression bags or bags with desiccating agents. In future studies, various hygroscopic medications will be tested for safe storage in one-dose packaging bags in clinical settings.

## Conclusions

In conclusion, the moisture-suppression bag effectively stored and preserved hygroscopic medications. The bag was more effective in inhibiting moisture absorption than plastic bags with desiccating agents under high temperature and humidity conditions. Moreover, the bag would be suitable for older adults because there is no fear of mistakenly consuming the desiccating agent. We expect that the moisture-suppression bag will improve patient convenience and therapeutic outcomes.

## Data Availability

The data that support the findings of this study are available from the corresponding author upon reasonable request.

## Abbreviations

RH: Relative humidity

## References

[1] Hasegawa K, Kuritani Y, Adachi A, Shinke K, Nishii S, Fujita Y (2008). Improvement of drug compliance and pharmaceutical care-types of drug and dosing regimens desired by patients. Jpn J Pharm Health Care Sci..

[2] Nakai K, Yamamoto N, Kamei M, Fujita M (2009). The effect of one-dose package on medication adherence for the elderly care in Japan. Pharm Pract.

[3] Matsuo T, Sadzuka Y (2018). Preservation methods for one-dose packages of high-hygroscopic Glucobay® tablets. Jpn J Pharm Health Care Sci..

[4] Sato K, Inaoka N, Kodama Y, Muro T, Nakamura T, Sasaki H (2018). Influence of storage conditions after one-dose packaging on stability of magnesium oxide tablets. Yakugaku Zasshi J Pharm Soc Jpn.

[5] Ashida T, Ono Y, Tanaka E, Uesugi N, Muraoka M, Komasa Y (2010). Accidental swallowing and aspiration in seven cities of the Hanshin area. Emergency calls the fire department for three years from 2004. Jpn J Dysphagia Rehabil.

[6] Sakuyama A, Jinbu Y, Hayasaka J, Tukimura H, Inoue K, Hayashi H (2018). A case of chemical injury of the oral mucosa caused by a desiccant. J Jpn Oral Med.

[7] Matsuo T, Yoshida Y, Tomita T, Sadzuka Y (2020). Relative humidity and qualities of hygroscopic medications stored in different one-dose packaging papers. Heliyon..

[8] Uyama R, Karasawa G, Kamiya K, Sugawara M, Karasawa Y (2005). Gastric perforation caused by calcium oxide in an adult: a case report. Nihon Fukubu Kyukyu Igakkai Zasshi (J Abdom Emerg Med).

[9] Inoue M, Hayama Y, Ikeyama N, Umemoto G, Toyofuku A, Kikuta T (2007). A case of schizophrenia with chemical burn of the oral mucosa caused by calcium oxide. J Jpn Stomatol Soc.

[10] Matsuo T, Sadzuka Y (2019). Appropriate evaluation of one-dose packaging of hygroscopic tablets: effects of combinations and number of hygroscopic tablets stored in one-dose packages on the retention period of medicines. Jpn J Pharm Health Care Sci.

